# Supplementary material for: Holm Oak (Quercus ilex subsp. ballota (Desf.) Samp.) Bark Aqueous Ammonia Extract for the Control of Invasive Forest Pathogens
Source: Int J Mol Sci. 2022 Oct 6;23(19):11882. doi: 10.3390/ijms231911882 (PMC9569932; doi:10.3390/ijms231911882)
Supplement: Supplementary file 1 [file ijms-23-11882-s001.zip › ijms-1950041-supplementary.pdf]

Holm Oak (*Quercus ilex* subsp. *ballota* (Desf.) Samp.) Bark Aqueous Ammonia Extract for the Control of Invasive Forest Pathogens

E. Sánchez-Hernández, J. Balduque-Gil, J.J. Barriuso-Vargas, J. Casanova-Gascón,
V. González-García, J.A. Cuchí-Oterino, B. Lorenzo-Vidal, J. Martín-Gil and P. Martín-Ramos

SUPPLEMENTARY MATERIAL

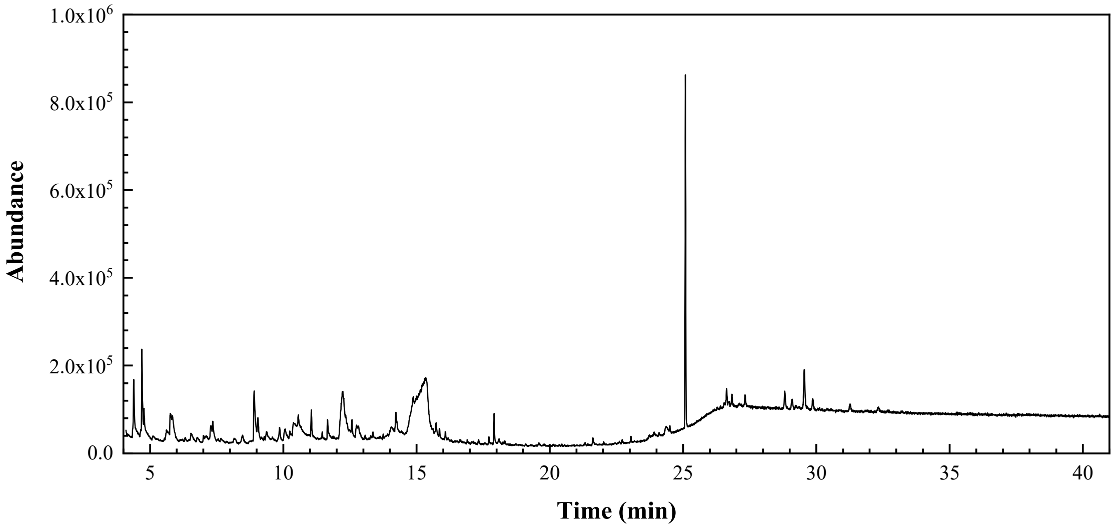

Figure S1. GC–MS chromatogram of *Q. ilex* subsp. *ballota* bark aqueous ammonia extract.

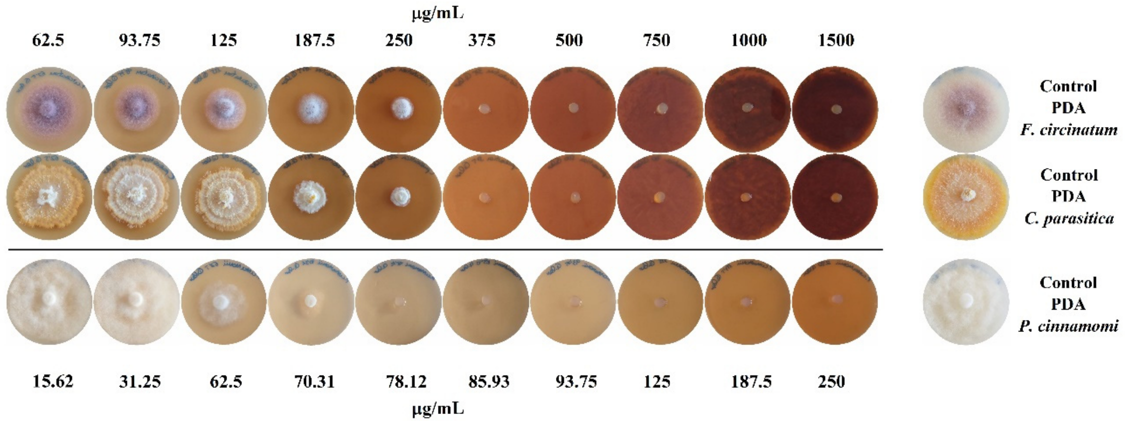

Figure S2. Mycelial growth inhibition of *F. circinatum* (top), *C. parasitica* (center), and *P. cinnamomi* (bottom) upon treatment at different concentrations with *Q. ilex* subsp. *ballota* bark extract. Only one replicate is shown. The control plates (PDA-only medium, without any amendments) are shown on the leftmost column.

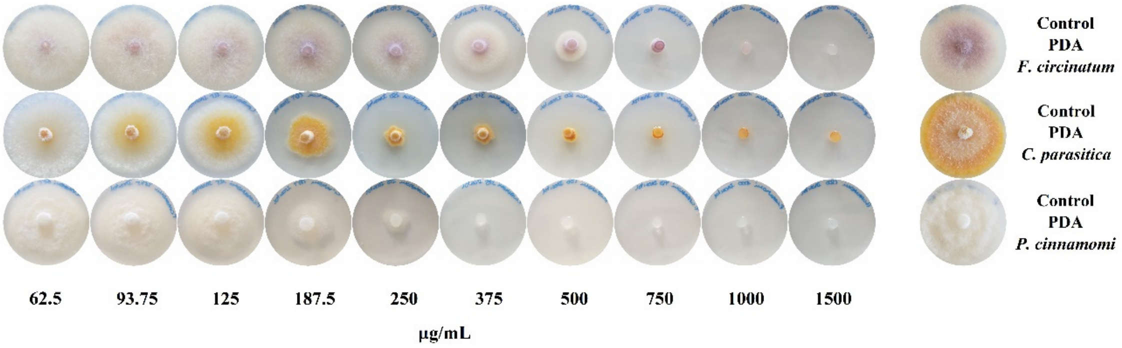

Figure S3. Mycelial growth inhibition of *F. circinatum* (top), *C. parasitica* (center), and *P. cinnamomi* (bottom) upon treatment at different concentrations with *myo*-inositol. Only one replicate is shown. The control plates (PDA-only medium, without any amendments) are shown on the leftmost column.

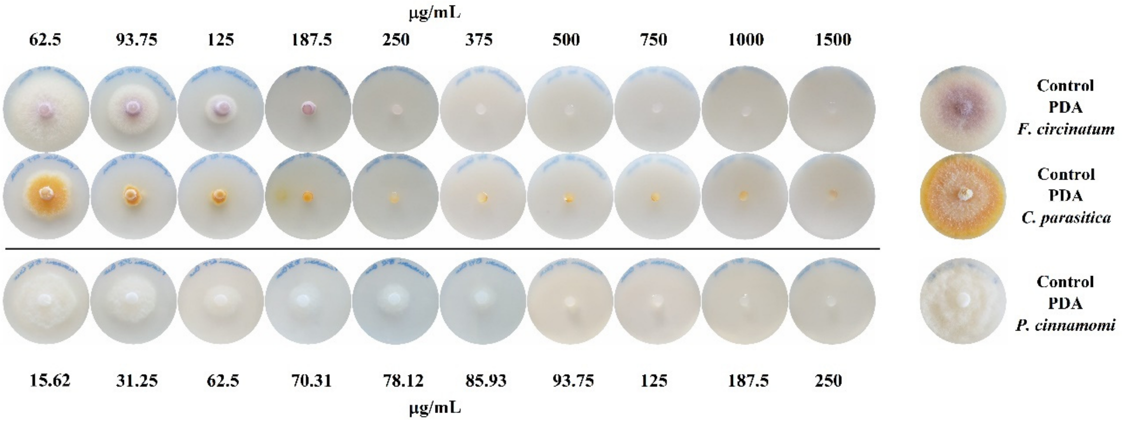

Figure S4. Mycelial growth inhibition of *F. circinatum* (top), *C. parasitica* (center), and *P. cinnamomi* (bottom) upon treatment at different concentrations with *trans*-squalene. Only one replicate is shown. The control plates (PDA-only medium, without any amendments) are shown on the leftmost column.

Table S1. Minimum inhibitory concentration (MIC) values reported in the literature for *Q. ilex* extracts against microorganisms.

Collection site	Extraction procedure	Microorganisms	MIC (μg·mL^{-1})	Other activity studied	Ref.
Extremadura (Spain)	Leaf ethanol or water extract (90%, *v/v*)	*Candida boidinii* CECT 11153	MIC = 2000	Antioxidant and antihypertensive activity	[1]
		Priceomyces carsonii CECT 10230	MIC = 2000		
		Kregervanrija fluxuum CECT 12787	MIC = 2000		
		Zygosacharomyces bailii CECT 11043	MIC = 2000		
Ourika (Morocco)	Bark ethyl acetate, butanol or water extracts (1.5, 16.05, 16.36%, *w/w*)	*Escherichia coli* ATCC 11775	MIC = 256-512	n.e.	[2]
		Pseudomonas aeruginosa ATCC 27853	MIC = 256-512		
		Staphylococcus aureus BCCM 21055	MIC = 128-256		
		Bacillus subtilis ATCC 6051	MIC = 128-256		
		Klebsiella pneumoniae ATCC 13883	MIC = 512		
		Salmonella typhimurium ATCC 43971	MIC = 256-512		
		Vibrio colerae ATCC 14033	MIC = 256-512		
		Proteus mirabilis HITM 20	MIC = 256-512		
		S. epidermidis HITM 60	MIC = 128-256		
		S. pyogenes HITM 100	MIC ≥ 512		
		S. agalactiae HITM 80	MIC ≥ 512		
Córdoba (Spain)	Leaf water extract (1:10)	*E. coli* CECT 8295	MIC = 2500	Effects of simulated gastrointestinal conditions on phenol stability and antioxidant activity	[3]
		Listeria monocytogenes CECT 4032	MIC = 5000		
		S. enterica subsp. *enterica* serovar Typhimurium CECT 704	MIC = 2500		
		S. aureus CECT 5193	MIC = 1000		
		Yersinia enterocolitica CECT 754	MIC = 1000		
Marmara region (Turkey)	Leaf methanol extract	*B. atrophaeus*	MIC = 250	n.e.	[4]
		B. cereus	MIC = 250		
		B. macerans	MIC = 250		
		B. pumilus	MIC = 250		
		B. sphaericus	MIC = 250		
		B. substilis	MIC = 125		
		Brucella abortus	MIC = 250		

		B. melitensis	MIC = 250		
		Enterobacter pyrinus	MIC = 250		
		E. intermedius	MIC = 250		
		E. coli	MIC = 250		
		Neisseria spp.	MIC = 250		
		P. fluorescens	MIC = 250		
		P. putida	MIC = 250		
		P. syringae	MIC = 250		
		C. albicans	MIC = 125		
Fez (Morocco)	Pollen ethanol extract (50%)	*S. aureus* 20s1	MIC = 620	Antioxidant activity	[5]
		Acinetobacter baumannii 118e1	MIC = 310		
		E. cloacae 57e2/n	MIC = 1250		
		E. coli 7	MIC = 1250		
		K. pneumoniae 6	MIC = 2500		
		P. aeruginosa 8e1	MIC = 2500		

n.e. = not specified.

Table S2. Antimicrobial activity reported in the literature for other natural products rich in *trans*-squalene.

Natural product	Content (%)	Activity	MIC/EC_{90} (μg·mL^{-1})	Ref.
Q. ilex subsp. *ballota* bark extract	13	*F. circinatum*	MIC = 250	This work
		C. parasitica	MIC = 187.5	
		P. cinnamomi	MIC = 93.75	
Ocimum basilicum leaves	1.94	*Bipolaris ellisii* CBS 19362	MIC = 32,000	[6]
		B. hwaiensis AUMC 1120	MIC = 16,000	
		B. spicifera AUMC 459	MIC = 32,000	
Mentha piperita	11.6	*B. cereus*	MIC = 70	[7]
		S. aureus	MIC = 60	
		L. monocytogene	MIC = 90	
		E. coli	MIC = 110	
		K. pneumonia	MIC = 100	
		S. typhi	MIC = 120	
		C. gelbeta	MIC = 120	
		C. tropicalis	MIC = 95	
		C. albicans	MIC = 80	
		A. niger	MIC = 150	
		A. fumigatus	MIC = 65	
		A. flavus	MIC = 110	
O. basilicum	9.2	*B. cereus*	MIC = 110	
		S. aureus	MIC = 100	
		L. monocytogene	MIC = 130	
		E. coli	MIC = 140	
		K. pneumonia	MIC = 130	
		S. typhi	MIC = 160	
		C. gelbeta	MIC = 140	
		C. tropicalis	MIC = 120	
		C. albicans	MIC = 110	
		A. niger	MIC = 170	
		A. fumigatus	MIC = 90	
		A. flavus	MIC = 125	
Lavandula	7.7	*B. cereus*	MIC = 100	
		S. aureus	MIC = 90	
		L. monocytogene	MIC = 120	
		E. coli	MIC = 130	
		K. pneumonia	MIC = 120	
		S. typhi	MIC = 150	
		C. gelbeta	MIC = 130	
		C. tropicalis	MIC = 110	
		C. albicans	MIC = 90	
		A. niger	MIC = 160	
		A. fumigatus	MIC = 75	
		A. flavus	MIC = 120	
Cymbopogon citratus	10.2	*B. cereus*	MIC = 80	
		S. aureus	MIC = 70	
		L. monocytogene	MIC = 100	
		E. coli	MIC = 120	
		K. pneumonia	MIC = 110	

		S. typhi	MIC = 140	
		C. gelbeta	MIC = 110	
		C. tropicalis	MIC = 85	
		C. albicans	MIC = 65	
		A. niger	MIC = 130	
		A. fumigatus	MIC = 50	
		A. flavus	MIC = 100	
Acacia auriculiformis leaves	n.e.	*Xanthomonas oryzae* pv. *oryzae*	MIC = 3130	[8]
A. mangium leaves	n.e.	*X. oryzae* pv. *oryzae*	MIC = 1560	
Rubia tinctorum roots	0.35	*Diplodia seriata*	EC_{90} = 87.8	[9]
		Dothiorella viticola	EC_{90} = 90.2	
		Neofusicoccum parvum	EC_{90} = 184.0	

References (reference numbers do not match those in the main text)

1. Boy, F.R.; Casquete, R.; Martínez, A.; Córdoba, M.d.G.; Ruíz-Moyano, S.; Benito, M.J. Antioxidant, antihypertensive and antimicrobial properties of phenolic compounds obtained from native plants by different extraction methods. *International Journal of Environmental Research and Public Health* **2021**, *18*, 2475.
2. Berahou, A.; Auhmani, A.; Fdil, N.; Benharref, A.; Jana, M.; Gadhi, C.A. Antibacterial activity of *Quercus ilex* bark's extracts. *J. Ethnopharmacol.* **2007**, *112*, 426-429, doi:10.1016/j.jep.2007.03.032.
3. Sánchez-Gutiérrez, M.; Gómez-García, R.; Carrasco, E.; Bascón-Villegas, I.; Rodríguez, A.; Pintado, M. *Quercus ilex* leaf as a functional ingredient: Polyphenolic profile and antioxidant activity throughout simulated gastrointestinal digestion and antimicrobial activity. *Journal of Functional Foods* **2022**, *91*, doi:10.1016/j.jff.2022.105025.
4. Güllüce, M.; Adıgüzel, A.; Öğütçü, H.; Şengül, M.; Karaman, İ.; Şahin, F. Antimicrobial effects of *Quercus ilex* L. extract. *Phytotherapy Research* **2004**, *18*, 208-211, doi:10.1002/ptr.1419.
5. Bakour, M.; Laaroussi, H.; Ousaaid, D.; Oumokhtar, B.; Lyoussi, B.; Romeo, F.V. Antioxidant and antibacterial effects of pollen extracts on human multidrug-resistant pathogenic bacteria. *Journal of Food Quality* **2021**, *2021*, 1-11, doi:10.1155/2021/5560182.
6. Elsherbiny, E.A.; Safwat, N.A.; Elaasser, M.M. Fungitoxicity of organic extracts of *Ocimum basilicum* on growth and morphogenesis of *Bipolaris* species (teleomorph *Cochliobolus*). *Journal of Applied Microbiology* **2017**, *123*, 841-852, doi:10.1111/jam.13543.
7. El-Saadony, M.T.; Saad, A.M.; Elakkad, H.A.; El-Tahan, A.M.; Alshahrani, O.A.; Alshilawi, M.S.; El-Sayed, H.; Amin, S.A.; Ahmed, A.I. Flavoring and extending the shelf life of cucumber juice with aroma compounds-rich herbal extracts at 4 °C through controlling chemical and microbial fluctuations. *Saudi Journal of Biological Sciences* **2022**, *29*, 346-354, doi:10.1016/j.sjbs.2021.08.092.
8. Shafiei, S.N.S.; Ahmad, K.; Ikhsan, N.; Ismail, S.I.; Sijam, K. Antibacterial activity of *Acacia* spp . Leaves extracts against *Xanthomonas oryzae* pv . *oryzae* and screening for active phytochemical contents. *IOSR Journal of Agriculture and Veterinary Science* **2017**, *10*, 49-60.
9. Langa-Lomba, N.; Sánchez-Hernández, E.; Buzón-Durán, L.; González-García, V.; Casanova-Gascón, J.; Martín-Gil, J.; Martín-Ramos, P. Activity of anthracenediones and flavoring phenols in hydromethanolic extracts of *Rubia tinctorum* against grapevine phytopathogenic fungi. *Plants* **2021**, *10*, doi:10.3390/plants10081527.
